# An effective method for ecosystem‐scale manipulation of bird abundance and species richness

**DOI:** 10.1002/ece3.5509

**Published:** 2019-08-13

**Authors:** Chelsea L. Wood, Margaret Summerside, Pieter T. J. Johnson

**Affiliations:** ^1^ School of Aquatic and Fishery Sciences University of Washington Seattle Washington; ^2^ Department of Ecology and Evolutionary Biology University of Colorado at Boulder Boulder Colorado

**Keywords:** avian diversity, cage, California, deterrent, exclusion, freshwater, pond, wetland

## Abstract

Manipulation experiments are a cornerstone of ecological research, but can be logistically challenging to execute—particularly when they are intended to isolate the ecological role of large, vagile species, like birds. Despite indirect evidence that birds are influential in many ecosystems, large‐scale, multi‐year bird manipulation experiments are rare. When these studies are conducted, they are typically realized with caged or netted exclosures, an approach that can be expensive, risky for wildlife, and difficult to maintain. In cases where caged exclosures are not appropriate, alternate approaches are needed to allow rigorous empirical studies on the ecological role of birds. Here, we present and validate a method for experimentally increasing the abundance and richness of birds at the scale of entire aquatic ecosystems. Unlike bird exclusion, this approach is experimentally tractable, appealing to land managers, and possible to deploy over large spatial scales. We tested the efficacy of our approach for increasing bird abundance and species richness at 16 central California ponds. Based on bird visitation data obtained by summer camera trapping, our approach significantly increased bird species richness and abundance at manipulated ponds compared to control ponds. Attractant treatments mitigated the negative effects of a major drought on bird species richness and generated a near doubling of bird abundance in the presence of attractants. Treatments had no effect on most mammal species, with the exception of ground squirrels, which increased in abundance in the presence of attractants. These results suggest that attractants are effective in increasing bird abundance and richness. We encourage researchers to consider this approach for experimentally isolating the ecological role of birds in aquatic and open terrestrial ecosystems, especially in cases where cost or logistical constraints preclude the use of caged or netted exclosures.

## 1 INTRODUCTION

Manipulation experiments have driven tremendous progress in ecology. Correlational and comparative work can point to potential ecological roles for particular taxa, but experimental manipulations are needed to understand causal relationships (Lubchenco & Real, 1991). Although the spatial extent of a manipulative field experiment will often be limited by logistical constraints, creative approaches have allowed ecologists to conduct these studies at the scale of entire ecosystems, including islands (e.g., Calsbeek & Cox, 2010; Simberloff & Wilson, 1969; Wilson & Simberloff, 1969), lakes (e.g., Carpenter et al., 1987), and forest watersheds (e.g., Likens, Bormann, Johnson, Fisher, & Pierce, 1970). Whole‐ecosystem manipulations offer a powerful means of hypothesis testing within complex, real‐world systems and have fundamentally influenced ecological thought on such topics as species–area curves, nutrient limitation in aquatic systems, trophic cascades, aquatic–terrestrial linkages, and the effects of invasive species. Here, we present a method for experimentally increasing the abundance and richness of birds at the scale of wetland ecosystems.

Although the ecological influence of birds is frequently overlooked, especially in aquatic systems, several studies point to their importance in influencing food web structure (Sekercioglu, 2006); recent anthropogenic declines in bird abundance make understanding their ecological role an urgent priority (Brooks et al., 2002; Hobbs & Mooney, 1998; Ziolkowski, Pardieck, & Sauer, 2010). In some stream, pond, and lake ecosystems, birds regulate the abundance of fish, which are often assumed to function as the apex predators in such environments (e.g., Beckmann, Biro, & Post, 2006; Crowder, Squires, & Rice, 1997; Matkowski, 1989; Myers & Peterka, 1976; Steinmetz, Kohler, & Soluk, 2003; Wood, 1987). Birds can also function as key nutrient importers in some habitats, including lakes (Manny, Johnson, & Wetzel, 1994), islands (Croll, Maron, Estes, Danner, & Byrd, 2005; Stapp, Polis, & Sanchez Pinero, 1999; Young, McCauley, Dunbar, & Dirzo, 2010), urban forests (Fujita & Koike, 2007), and intertidal ecosystems (Bosman, Toit, Hockey, & Branch, 1986). Beyond their direct effects, birds also function as a transport system for other organisms, both free‐living and parasitic. Birds are common definitive hosts for many trophically transmitted parasites and can serve as a major source of trematode, tapeworm, nematode, and acanthocephalan larvae to aquatic habitats (Bush, 1989; Poulin, 1995), where the parasites may then infect frogs, fish, and benthic invertebrates (e.g., Hechinger & Lafferty, 2005; Johnson, Sutherland, Kinsella, & Lunde, 2004). Other ecological roles for birds include seed dispersal, pollination, scavenging, and pest control (Sekercioglu, 2006).

Progress in understanding the ecological role of birds at the ecosystem level has been hampered by the difficulty of performing experimental manipulations of bird abundance across large spatial extents (Sekercioglu, 2006; Table 1). Birds are highly vagile, meaning that bird exclusions, once cleared of birds, must be carefully protected against immigration of new individuals. Some birds are also sensitive wildlife species; exclusion experiments must minimize the risk of injury to birds, displacement of birds from critical habitat, and other potentially deleterious effects on bird populations. Exclosures that rely on caging or netting are subject to a variety of additional constraints, including wildlife entanglement risks, the cost and labor required for establishment and maintenance, and the risk of exclosure failure due to wear‐and‐tear, vandalism, and weather events. Use of caged or netted exclosures therefore often places a strict upper limit on the spatial and temporal scope of an experiment. Another option is individually repelling birds that attempt to approach exclosure zones, using human presence and disturbance. In one study, researchers repelled gulls from large (530–1,152 m^2^) sections of the rocky intertidal zone over the course of 26 days by shooting birds with streams of water from “supersoaker” water guns (Ellis, Shulman, Wood, Witman, & Lozyniak, 2007). However, this approach is extremely labor‐intensive and therefore limits the temporal duration of an experiment. Large‐scale (Englund, 1997) and long‐term (Bender, Case, & Gilpin, 1984) experimental units are important for achieving biological realism and avoiding confounds in exclusion experiments. Constraints on the spatial and temporal scope of such experiments therefore substantially diminish ecologists' ability to explore the ecological role of birds in food webs.

**Table 1:** Examples of studies in which birds were excluded from habitat using cages or netting. This list is not exhaustive and does not include studies in which individual plants were protected against birds. Entries are organized by area and duration of exclosure. Studies referenced in Notes, below

Study	Habitat exclosed	Area exclosed	Duration of exclosure	Goal of study
1	Bahamian islands	800 m^2^ to 2,300 m^2^	4 months	Test whether bird (and reptile) predation affects natural selection in lizards
2	Perennial grassland	30.5‐m × 15.2‐m	3 years	Test whether predation by insectivorous birds can control grasshopper populations and assess the effects of such control on grassland community composition
3	Stream reaches	12‐m wide × 60‐m long	2 months	Test whether predation by herons and kingfishers can affect the abundance and size of fish
4	Deciduous forest	15‐m × 15‐m	~2 months	Test whether predation by great tits and nuthatches can control insect abundance and assess the effects of such control on tree leaf damage
5	Deciduous forest	6‐m x 6‐m	~2 months	Test whether predation by insectivorous birds can control leaf‐eating insect abundance
6	Hawaiian forest	4–6‐m × 4–6‐m	32 months	Test whether predation by insectivorous birds can control insect abundance and assess the effects of such control on tree growth
7	Shallow lake	4‐m × 5‐m	~4 months	Test whether herbivory by waterfowl can control abundance of submerged vegetation and macroinvertebrate biomass
8	Intertidal mudflat	2‐m × 2‐m	~10 months	Test whether predation by shorebirds can control abundance of benthic invertebrates
9	Boreal forest	2‐m × 2‐m	~2.5 months	Test whether predation by insectivorous birds can control insect abundance and assess the effects of such control on plant shoot damage
10	Intertidal mudflat	1‐m × 1‐m	~1 year	Test whether predation by shorebirds can control abundance of benthic invertebrates
11	Freshwater wetland	1‐m × 1‐m	3 months	Test whether predation by aquatic birds can control abundance and biomass of benthic invertebrates
12	Rocky intertidal	1‐m × 1‐m	~3 weeks	Test whether crabs can control abundance of snails in the absence of predation by gulls
13	Rocky intertidal	49‐cm × 39‐cm	~2 years	Test whether predation by birds can control the abundance of urchins and abundance and diversity of macro‐algae
14	Rocky intertidal	29‐cm × 34‐cm	Variable, from 9 to 87 days	Test whether predation by birds can control intertidal community composition

Note

1. Calsbeek and Cox (2010), 2. Bock, Bock, and Grant (1992), 3. Steinmetz et al. (2003), 4. Murakami and Nakano (2000), 5. Holmes, Schultz, and Nothnagle (1979), 6. Gruner (2004), 7. Marklund, Sandsten, Hansson, and Blindow (2002), 8. Raffaelli and Milne (1987), 9. Atlegrim (1989), 10. Quammen (1984), 11. Ashley, Robinson, Oring, and Vinyard (2000), 12. Ellis et al. (2007), 13. Wootton (1995), 14. Wootton (1993).

Treatments that increase bird abundance are an alternative to exclusion treatments and might lift some of these constraints on spatial and temporal scope. Although ecologists have identified many factors that promote bird visitation to sites and even augment bird populations (e.g., Blewett & Marzluff, 2005; Donnelly & Marzluff, 2006; James & Wamer, 1982; MacArthur & MacArthur, 1961; Rotenberry, 1985; Roth, 1976), this knowledge has been infrequently applied to ecological experimentation. The few experiments that have been conducted augment bird population size at small spatial extents (e.g., Athie & Dias, 2016; Smith, 2001; Wolff, Fox, Skillen, & Wang, 1999), rather than at the ecosystem level. Although augmentation experiments cannot typically assess the implications of a species’ absence in the manner achieved by exclusion studies, they nonetheless provide quantitative estimates of a species' effect along a gradient in its abundance. Importantly, in ecosystems that are already degraded, augmentation treatments might simulate the natural state of the ecosystem. Finally, exclusion treatments that limit bird access to a site also limit access by co‐occurring taxa, like mammals and reptiles; in order to isolate the influence of birds, treatments must change only bird abundance, an approach that can be achieved with augmentation treatments (e.g., Athie & Dias, 2016; Smith, 2001; Wolff et al., 1999).

Here, we propose a method that allows researchers to experimentally increase bird abundance and richness over large spatial extents, with minimal cost, risk to wildlife, and need for maintenance. This approach involves the use of attractants that encourage birds to use a particular site, instead of deterrents that discourage birds from using that site (an approach whose efficacy often attenuates over time) or physically preventing their access to the site. With a combination of enhanced nesting, roosting, and perching habitat, we demonstrate the efficacy of this approach in increasing the abundance and species richness of water‐associated birds at central California ponds, while leaving the abundance of most other co‐occurring large vertebrate taxa unchanged. We anticipate that this method could be effectively applied to other freshwater ecosystems, including ponds, lakes, and streams, as well as estuarine and open terrestrial ecosystems.

## 2 MATERIALS AND METHODS

### 2.1 Sites and study design

**Figure 1:**
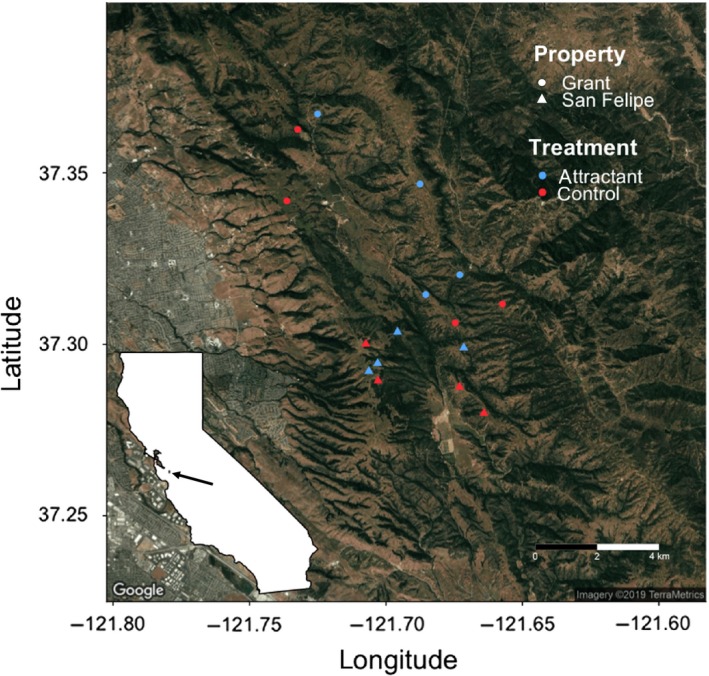
Map of study sites in the East Bay region of central California. Eight experimental ponds were located in Joseph D. Grant County Park (circles) and eight were located on San Felipe Ranch (triangles). Of these, eight were randomly assigned to the bird attractant treatment (blue) and eight were randomly assigned to the control treatment (red)

We selected 16 small ponds located on two adjoining properties in the East Bay area of central California (Figure 1). This area is located on the Pacific flyway, which serves as one of four major migration routes for birds in North America and provides naturally high levels of bird activity (Migratory Bird Program 2012). We selected eight ponds at Joseph D. Grant County Park and another eight at San Felipe Ranch, based on accessibility and existence of prior data. All ponds were then randomly assigned to one of two treatments: attractant or control (eight ponds per treatment, four on each property). The ponds were all at least ~1 km apart and occur in oak woodland habitat typical of the California Floristic Province.

**Figure 2:**
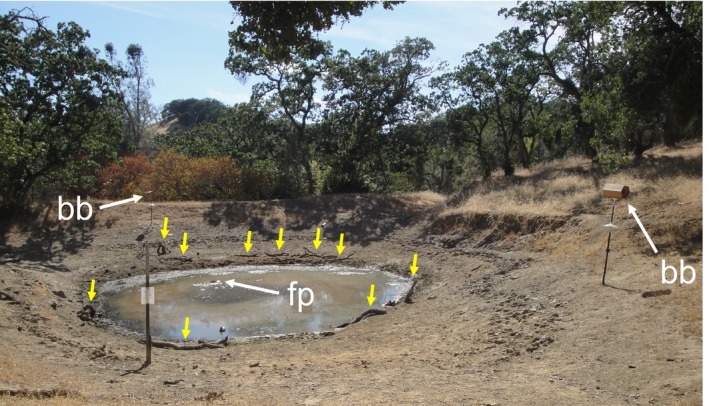
Attractant manipulations installed at Glorious Pond, Joseph D. Grant County Park. bb = bird nesting boxes, fp = floating platforms, yellow arrows indicate added perching habitat

To attract birds to attractant‐treatment sites, we added perching habitat, nesting habitat, two mallard duck decoys (one male, one female), and one floating platform to each pond (Figure 2). Any natural perching habitat that we found to be available in the vicinity of the pond, such as coarse woody debris (branch diameter range ~1 cm–15 cm), was haphazardly distributed closer to the water's edge. If no perching habitat was available, we brought branches in from the nearby forest. One wood duck nesting box and one generic bird nesting box (Backyard Boys Woodworking, Green Bay, WI) were installed at each site by mounting on 6‐foot fence posts equipped with a predator guard. We constructed floating platforms using wooden pallets and sealed, 1.5‐inch PVC‐pipe floats and anchored one platform to the bottom of each pond, with a slackline to allow for rising and falling pond water levels. Previous research has demonstrated the value of adding perching habitat (e.g., Dickson, Conner, & Williamson, 1983; Kay, Twigg, Korn, & Nicol, 1994; McClanahan & Wolfe, 2002; Smith, 2001), nest boxes (e.g., Newton, 1994), bird decoys (e.g., Crozier & Gawlik, 2003), and floating platforms (Davis & Jackson, 2000; Piper, Meyer, Klich, Tischler, & Dolsen, 2002; Shealer, Buzzell, & Heiar, 2006) for bird conservation. All manipulations were installed in June and early July 2015.

### 2.2 Assessment of bird visitation to ponds

**Figure 3:**
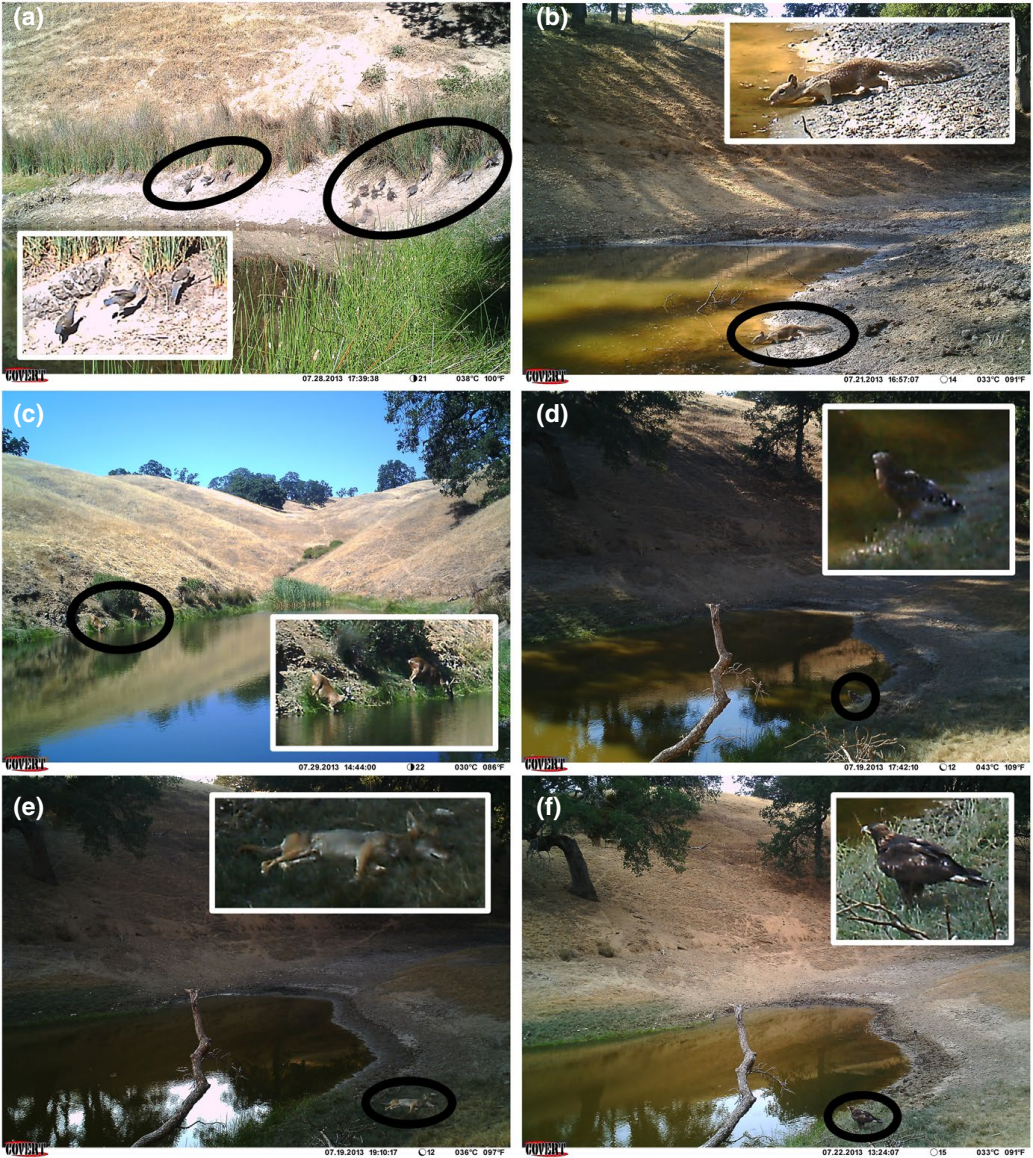
Photographs taken by trail cameras. (a) California Quails in narrow view, (b) ground squirrel in narrow view, (c) deer in broad view, (d) Red‐shouldered Hawk in broad view, (e) coyote in broad view, (f) Golden Eagle in broad view

We assessed bird abundance by monitoring ponds with DLC Covert MP6 trail cameras (Covert Scouting Cameras, Inc.). Cameras were mounted and secured to lengths of rebar driven into the pond shoreline and were set to capture one image every three minutes during daylight hours. Two cameras were installed at each pond: One camera (the “broad” camera) was trained on a large section of the pond to capture images of large, rare birds (e.g., raptors), and mammals (e.g., deer, pigs, coyotes, cows), while the second camera (the “narrow” camera) was mounted at the water–shoreline interface and trained on the immediately adjacent shoreline, to capture higher‐resolution images of smaller, more common birds (e.g., jays, phoebes, quails) and mammals (e.g., ground squirrels). Cameras were consistently placed in the same location for each round of camera trapping. This dual approach allowed us the spatial coverage to capture rare species while permitting the image resolution to reliably identify smaller, common species (Figure 3), which was tractable given the small size of the ponds included in this study (range = 26–4,923 m^2^, mean = 519 m^2^). Each bird and mammal species was quantified using only one image type (i.e., broad or narrow) to avoid double counting. We made the broad versus narrow selection for each bird species by counting the total number of observations of birds across all photos taken by each camera type and choosing the camera type that yielded the greatest number of observations for each bird species. Bird species richness was summed between the two camera types to obtain total richness across all birds for each site–day combination. We set cameras to capture photographs in one sampling bout one year prior to installation of treatments (3–9 July 2014; hereafter, “before”) and a second sampling bout two years after installation of treatments (1–8 July 2017; hereafter, “after”). We chose to monitor the long‐term response of bird abundance and richness to treatments for several reasons: (a) birds might need time to acclimate to new stimuli in the environment, (b) effects of attractant treatments could decay through time, and (c) because we sought to evaluate treatment efficacy across the time scales likely to be used in future bird manipulation experiments designed to evaluate the ecological roles of birds.

At the end of each deployment, images from cameras were downloaded and photographs scored in Timelapse Image Analyzer (Saul Greenberg, University of Calgary, Calgary, Alberta). We excluded photographs taken in the first and last five minutes of deployment, while a researcher was present at the pond for camera collection, during rainy conditions, and when glare compromised images. For the remaining photographs, each visible bird and mammal was marked in the Timelapse Image Analyzer program, identified to species, and counted. Where a species‐level identification was not possible, birds were grouped as “unidentified passerines.” We grouped some species (e.g., Mallards [*Anas platyrhynchos*], which were common, with Great Blue Herons [*Ardea herodias*] and Black Brant [*Branta bernicla nigricans*], which were not) to facilitate analysis.

### 2.3 Experimental design

We compared the change in bird richness and abundance from before the manipulation (i.e., 2014) to two years after manipulation (i.e., 2017) in control versus attractant treatments (a before–after–control–impact or BACI design). Trail cameras were deployed for 4–8 days (average = 6.7 days) during each evaluation period; we calculated bird species richness and bird visitation for each deployment day, and replicates in the analysis therefore represent the number of bird taxa or individuals observed per deployment day. We excluded any site–day where the number of photos taken was fewer than 20 (i.e., <1 hr of observation; average number of photos per day = 185 or 9.3 hr of observation). Summing within each day helped to alleviate the zero‐inflation problem inherent to a dataset that contains many photos with no birds and de‐emphasized circadian/diurnal patterns, which were not the focus of the study.

### 2.4 Statistical analysis

We used two approaches to estimate bird species richness: raw richness (raw number of species observed per day) and the non‐parametric jackknife estimator of species richness. We included the jackknife estimate to project bird species richness at the saturation of the species accumulation curve for each date at each pond and calculated it using the SPECIES package in R (Wang, 2011). This approach produces an estimate of richness that is independent of estimates of abundance (i.e., the approach corrects for the fact that number of birds observed or number of photographs scored might influence the estimate of richness; Gotelli & Colwell, 2001). For site–day combinations in which the jackknife estimate failed to converge (i.e., for site–days where there were too few bird detections to calculate bird species richness at the saturation of the species accumulation curve), we excluded that site–day. The number of photographs taken varied among deployment days; for example, if a camera was retrieved in the morning, only a few photographs would be recorded from that final day of deployment. To correct for this unevenness in effective sampling effort, we included number of photographs taken per deployment as a covariate in our analysis when analyzing abundance, but not for raw richness (because sampling effort and raw richness are likely to be related non‐linearly; Gotelli & Colwell, 2001) or the jackknife estimate of richness (because the jackknife estimate inherently corrects for differences in sampling effort).

To assess the impact of the attractant treatment on raw bird species richness and the jackknife estimate of bird species richness, we used a BACI framework, running generalized linear mixed‐effects models with a fixed effect of treatment (i.e., control, attractant), a fixed effect of time (i.e., before manipulation [2014], after manipulation [2017]), an interaction term (treatment*time), a fixed effect of property (i.e., Grant County Park or San Felipe Ranch), and a random effect of pond identity (to account for multiple observations at each pond):

$$\begin{array}{c} \left({\text{RawBirdSpeciesRichness}}_{\mathrm{ij}}\right)\;\text{or}\;\left({\text{JackknifeEstimateOfBirdSpeciesRichness}}_{\mathrm{ij}}\right)∼\\ {\text{Treatment}}_{\mathrm{ij}}*{\text{Time}}_{\mathrm{ij}}+{\text{Property}}_{\mathrm{ij}}+\left(\left.1\right|{\text{PondIdentity}}_{i}\right), \end{array}$$

where the response variable*_ij_* represents the *j*th day of observation in pond *i*. Analyses were conducted using the *glmer()* function in the lme4 package in R (Bates, Maechler, Bolker, & Walker, 2015) using Poisson error structure and a log‐link function.

We also used the BACI framework to assess the influence of treatments on bird visitation rates (i.e., daily bird abundance within ponds). We first summed bird abundance across all photographs within each day for each bird species (within the camera type [broad vs. narrow] used to quantify the abundance of that species). The generalized linear mixed‐effects model took the form,

$$\begin{array}{c} {\text{BirdAbundance}}_{\mathrm{ijk}}∼{\text{Treatment}}_{\mathrm{ijk}}*{\text{Time}}_{\mathrm{ijk}}+{\text{Property}}_{\mathrm{ijk}}+{\text{CameraType}}_{\mathrm{ijk}}+\\ \left(\left.1\right|{\text{PondIdentity}}_{i}\right)+\left(\left.1\right|{\text{BirdTaxon}}_{k}\right)+\text{offset}\left(\log\left[{\text{NumberOfPhotosTaken}}_{\mathrm{ij}}\right]\right), \end{array}$$

where the response variable*_ijk_* represents the *j*th day of observation in pond *i* for bird species *k*. Camera type indicates whether the broad or narrow camera view was used, and the random effect of bird taxon (i.e., species identity) controls for multiple observations within each bird taxon. To control for differences among site–days in effective sampling effort, we included an offset term for log[number of photos taken], which effectively converts the response variable to a rate (number of birds per photo). The response variable was modeled as a negative binomial distribution to account for overdisperson using the *glmer.nb()* function in the lme4 package in R (Bates et al., 2015).

To assess the influence of treatments on mammal (i.e., coyote, pig, deer, cow, ground squirrel) visitation rates, we used a generalized linear mixed‐effects model to analyze the number of mammals within each day. The generalized linear mixed‐effects model took the form,

$$\begin{array}{c} {\text{MammalAbundance}}_{\mathrm{ijk}}∼{\text{Treatment}}_{\mathrm{ij}}*{\text{Time}}_{\mathrm{ij}}*{\text{MammalSpecies}}_{\mathrm{ijk}}+{\text{Property}}_{\mathrm{ijk}}+\\ {\text{CameraType}}_{\mathrm{ijk}}+\left(\left.1\right|{\text{PondIdentity}}_{i}\right)+\text{offset}\left(\log\left[{\text{NumberOfPhotosTaken}}_{\mathrm{ijk}}\right]\right), \end{array}$$

where the response variable*_ij_* represents the *j*th day of observation in pond *i* for mammal species *k*. The response variable was modeled as a negative binomial distribution to account for overdisperson using the *glmer.nb()* function in the lme4 package in R (Bates et al., 2015).

## 3 RESULTS

**Figure 4:**
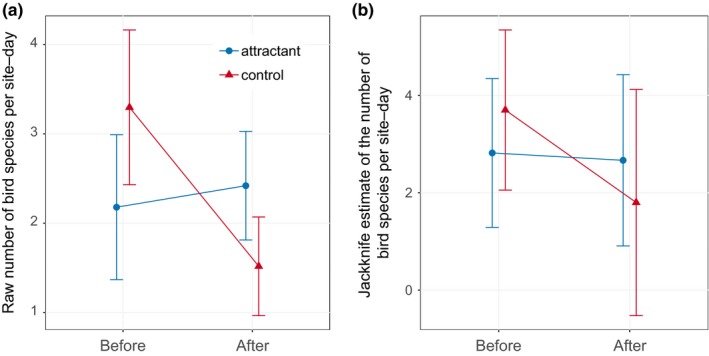
Mean (a) raw and (b) jackknife‐estimated bird species richness in the presence (blue) and absence (red) of bird attractants, before (2014) and two years after (2017) establishment of treatments. Error bars indicate 1 *SE*

In total, we detected 29 bird species and five mammal species. The bird detections were dominated by a few taxa (California Quail, Mallards, Stellar's Jays, Mourning Doves, and unidentified passerines), which together accounted for 91% of observations. Bird taxonomic richness declined over time in both treatments, but this decrease was less pronounced in the attractant compared to control treatments, both for raw richness (Poisson GLMM: treatment[control]*time[before] = coefficient ± 1 *SE* = +0.9018 ± 0.2511, *z* = 3.59, *n* observations = 115, *n* groups = 16, *p* = .0003; Figure 4a) and for the jackknife estimator of richness (NB GLMM: treatment[control]*time[before] = coefficient ± 1 *SE* = +1.2294 ± 0.5907, *z* = 2.08, *n* observations = 32, *n* groups = 13, *p* = .0374; Figure 4b). Effect sizes for the effect of the treatment by time interaction were modest; the implementation of attractant treatments resulted in the addition of 0.90 raw species and 1.23 jackknife‐estimated species between the before and after time points, relative to the control. Richness declined between 2014 and 2017 in the control treatment—probably due to the effects of a state‐wide drought that began in 2011 and intensified in 2015 (see below; Figure 4); this decline was mitigated by the attractant treatment. Losses in richness among the control ponds primarily involved American Robins, Black Phoebes, California Quail, Western Kingbirds, unidentified passerines, raptors, and waterbirds.

**Figure 5:**
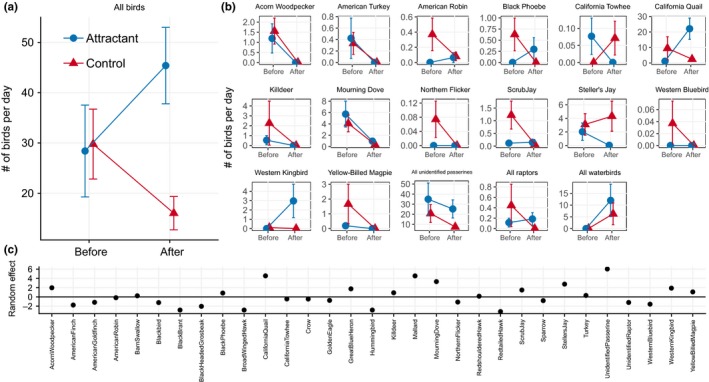
Abundance of birds in the presence (blue) and absence (red) of bird attractants, before (2014) and two years after (2017) establishment of treatments: (a) all birds and (b) individual bird species. Error bars indicate 1 *SE*. (c) Random effects of each bird taxon from GLMM of bird abundance

Bird abundance was also augmented by the attractant treatment. Total bird abundance (across all taxa) increased in the attractant treatment while it declined in the control treatment (NB GLMM: treatment[control]*time[before] = coefficient ± 1 *SE* = +1.4347 ± 0.4598, *z* = 3.12, *n* observations = 3,541, *n* sites = 16, *n* species = 32, *p* = .0018; Figure 5a). The bird taxa in which the attractant treatment had the most positive influence on abundance were American Robins, Black Phoebes, California Quail, Western Kingbirds, unidentified passerines, raptors, and waterbirds (Figure 5b); together, these taxa accounted for 83% of total bird–days counted (see Figure 5c, which indicates the deviation from mean abundance across all bird taxa). A few bird taxa responded negatively to attractant treatments, including California Towhees and Steller's Jays (Figure 5b); these taxa constituted 5% of total bird–days counted.

With respect to mammals, we found no effects of the treatments on the abundance of mule deer (*Odocoileus hemionus;* NB GLMM: treatment[control]*time[before] = coefficient ± 1 *SE* = +1.0660 ± 1.4400, *z* = +0.74, *n* observations = 539, *n* sites = 16, *n* species = 5, *p* = .46), wild pigs (*Sus scrofa;* NB GLMM: treatment[control]*time[before] = coefficient ± 1 *SE* = –1.8410 ± 1.4270, *z* = −1.29, *n* observations = 539, *n* sites = 16, *n* species = 5, *p* = .20), or coyotes (*Canis latrans*; NB GLMM: treatment[control]*time[before] = coefficient ± 1 *SE* = –1.3420 ± 2.1190, *z* = –0.63, *n* observations = 539, *n* sites = 16, *n* species = 5, *p* = .53). The presence of cows was primarily influenced by grazing management decisions (i.e., the pastures to which cows were given access by land managers during the trail camera deployments). Ground squirrel (*Otospermophilus beecheyi*) visitation rates were increased by attractants (NB GLMM: treatment[control]*time[before] = coefficient ± 1 *SE* = +6.5493 ± 1.5704, *z* = +4.17, *n* observations = 539, *n* sites = 16, *n* species = 5, *p* < .0001).

## 4 DISCUSSION

The bird attractant treatments we implemented were effective in augmenting the abundance and species richness of birds at eight central California ponds, even two years after the establishment of treatments. Attractant treatments had a positive influence on the number of bird species observed per day, mitigating the effects of a major drought and lessening the decline in bird richness that occurred at control ponds. Together, these results suggest that simple, inexpensive modifications to existing pond habitat can produce a substantial change in bird abundance and richness—suggesting a way forward for field experiments that can effectively assess the ecological role of birds.

Between 2012 and 2017, California experienced the worst drought in its historical record (Swain, 2015), and this probably influenced the outcome of our experiment. We interpret the observed reduction in both bird species richness and abundance in control ponds as a response to these drought conditions. Attractant treatments appeared to mitigate the negative effects of drought on bird visitation, even producing an increase in bird abundance across a time period that saw declining bird abundance in control treatments. Perhaps the drought‐induced death of vegetation (Small, Roesler, & Larson, 2018) increased predation risk at ponds, and attractant treatments mitigated the perceived or actual threat of predation by providing protected perching habitat. The drought might have led to actual reductions in the population density of birds (i.e., reflected in the decline in abundance observed at control ponds), or contributed to birds moving to alternative, more optimal habitat, including the ponds with installed attractants.

Treatment effects were driven by a handful of bird taxa that made up the majority (83%) of bird sightings. The abundance of American Robins, Black Phoebes, California Quail, Western Kingbirds, unidentified passerines, raptors, and waterbirds was positively influenced by attractants, driving effects on overall richness and abundance. Quail and raptors may be especially responsive to pond‐side cover, as they tend to use pond edge habitat, rather than perching on floating substrates or wading, which might provide more protection from mammalian predators. Raptors (Bildstein, Schelsky, & Zalles, 1998; Fuller, 1996; Sergio, Newton, & Marchesi, 2005) and waterbirds (Kushlan et al., 2002; Stralberg et al., 2011) are the focus of many conservation efforts, and our treatments effectively increased their abundance by providing perching habitat and cover—making the manipulation we propose both a conservation intervention and an effective, ethical means by which to measure the ecological role of these species in pond ecosystems.

Visitation rates of mammals were unaffected by the presence of attractants, except in the case of ground squirrels. Deer, pigs, and coyote had no significant response to the presence of attractants. Cow presence at the experimental ponds was broadly determined by herd management decision‐making (i.e., which pastures cows are permitted to access). Interestingly, ground squirrels had a strong positive response to attractants, perhaps because treatments enhanced cover at pond edges, where squirrels consume water, or because the augmented number and richness of birds signaled safety from predators. This positive effect of treatments on squirrel abundance could introduce a confounding factor into experimental designs in which bird attractants are deployed. We encourage researchers interested in using this bird manipulation approach to plan protocols for quantifying the effect of attractants on rates of mammal visitation. With some modifications (e.g., placing additional perching habitat within a pond rather than at its edge), this method could allow researchers fine‐scale control to perform taxon‐specific manipulations that affect only birds while leaving mammalian visitation rates unaffected.

In addition to unexpected effects on mammal visitation rates, a few additional caveats are worth noting. We monitored bird abundance and richness over summer months (July 2014 and 2017) in order to make before and after contrasts optimally comparable and reduce noise arising from seasonal variability in bird visitation rates. However, bird activity varies substantially through the seasons, and our results might have been different and might have reflected the behavior of different bird species had we chosen to monitor at other times of year (e.g., during winter migrations). Given the large differences observed between control and treatment ponds, we suspect these results would be robust to seasonal variation, but that suspicion remains to be tested. As noted above, attractant treatments might not always be a suitable substitute for exclusion treatments. Attractant treatments can indicate the direction and magnitude of the effect of a particular taxon on its community, but to quantify that taxon's total effect, individuals of that taxon must be removed completely. Some ecological processes might not be amenable to experimentation using attractant treatments; for example, any non‐linear process in which change accelerates as bird density declines (e.g., seed dispersal; Morales & Carlo, 2008) would probably not be well characterized by an attractant experiment. Nonetheless, attractant treatments remain a useful, practical, and inexpensive alternative to exclusion treatments for the exploration of some ecological processes.

The manipulations we implemented were inexpensive, easily maintained, and unobtrusive. We estimate that our attractant treatments cost approximately US$103 per pond ($60 for wood duck box, $25 for generic bird box, $2 for fence posts to mount bird boxes, $6 for duck decoys, $10 for materials to construct floating platform), and required fewer than two person‐hours to install. By comparison, a netted exclosure of equivalent size could cost thousands of US dollars per pond, given the expense of heavy‐duty bird netting and materials for an exclosure frame (PVC, rebar, or weather‐treated lumber). A netted exclosure would also require dozens of person‐hours to install. In addition to their low cost, our manipulations were durable and easily maintained: despite the presence of large mammals (e.g., deer, pigs, coyote, cows) that might trample or otherwise compromise attractants, we observed no negative wildlife interactions. Mammals—including livestock—were able to use ponds during deployment of attractants; this would be impossible with a netted exclosure. Manipulations required very minimal maintenance; we checked on ponds once per year and spent ~15 person‐minutes per pond per year re‐positioning floating platforms or duck decoys, supplementing shoreline perching habitat, or (for only one pond over the two‐year experiment) re‐mounting a fallen bird box. Importantly, the manipulations were unobtrusive and inconspicuous. This low visibility minimizes the chance that the treatments will be noticed by human visitors, reducing the likelihood of vandalism, theft, and objections by neighbors, park users, landowners, or land managers concerned about the aesthetic value of ponds. In fact, one of the land managers we worked with was enthusiastic about these manipulations, which she hoped would contribute to the conservation value of wetlands under her stewardship (K. Cotter, J. D. Grant County Park, *personal communication*). The low cost, ease of maintenance, inconspicuousness, and conservation benefits of our approach allowed us to maximize the size and number of manipulated ponds, increasing statistical power and biological realism.

There are numerous potential applications of our approach to manipulating bird abundance and richness. We plan to use this method to perform a large‐scale, long‐term bird manipulation experiment in central California ponds. Our aim is to quantify the effect of increases in local bird abundance and richness on the composition of pond communities, and particularly on the transmission of parasites within ponds. Birds play a variety of roles in these pond ecosystems: as dispersers of parasites (Bush, 1989; Poulin, 1995), predators of hosts (Erwin, 1996), and hosts for vectors and the pathogens they transmit (Kilpatrick, 2011). Manipulative experiments are therefore necessary to disentangle the potential effects of change in bird biodiversity on disease processes and to discover the net effect of bird biodiversity loss on the prevalence of disease in ponds. Our method of bird augmentation might also be useful for scientists working on other questions about the ecological roles of birds, or in other ecosystems. Most bird manipulation experiments to date have investigated the role of birds as predators using bird deterrence, and bird exclusion is a suitable approach for assessing the impacts of bird predation on community composition at small spatial scales. However, because our approach can be deployed across larger spatial scales than traditional caged or netted bird exclosures, it can also be used to investigate processes that occur at large spatial scales: for example, nutrient export/import, seed dispersal, and scavenging/decomposition. Our approach could also be easily adapted to augment birds across large plots in other relatively open ecosystems—for example, grasslands, meadows, open woodlands, tundra, marshes, wetlands, dunes, and beaches.

## 5 CONCLUSION

Our approach was effective in increasing the richness and abundance of birds in aquatic ecosystems. This method may be appropriate for researchers seeking to manipulate the richness and abundance of birds in a variety of open ecosystems and offers several advantages over traditional caged or netted exclosures, including lower cost, lower risk of negative wildlife interactions, lower risk of experiment failure due to damage, and—most importantly—the ability to conduct experiments at large spatial and temporal scales. We encourage other researchers to consider using our approach to manipulate bird richness and abundance in their own studies of the ecological roles of birds, where the use of caged or netted exclosures would not be appropriate.

## CONFLICT OF INTEREST

None declared.

## AUTHOR'S CONTRIBUTIONS

CLW and PTJJ conceived the study. CLW and MS collected the data. CLW and PTJJ developed the statistical approach. CLW performed the statistical analysis and wrote the first draft. All authors contributed to later versions of the manuscript.

## Data Availability

For each trail camera photo, counts of each bird species, site name, site treatment, date, and time: Dryad https://doi.org/10.5061/dryad.64c7s74.
